# Asphyxia in the Drowned, Etc.

**Published:** 1880-04

**Authors:** 


Cuts to Illustrate Asphyxia in the Drowned and in the Neonatus.—We agree fully with an esteemed correspondent that "it would have attracted more immediate attention to the great value of your (our) 'Speedy Method in Asphyxia' in its most important forms, had cuts been introduced to illustrate its application to the *drowned*, as well as the *neonatus;* as when appeals are made to the eye and intellect in that way the impression on the mind is stronger and more enduring." The cuts introduced in the article were not as satisfactory as we would have desired them to be, as they were copied from a leather manikin and not from life. Nevertheless, the idea intended to be illustrated by them can be readily utilized in all forms of asphyxia if they are carefully considered in connection with the text. The *Scientific American*, high authority in all matters appertaining to discoveries and printed illustrations, uses similar cuts in copying our article into its pages; and the London *Medical Times and Gazette*, the foremost medical periodical of the world, whilst copying the article approvingly, used no cuts or illustrations at all. Our first article on the subject was published a decade ago (1870), but then in a new journal of limited circulation; and five years later, when further knowledge of its value became to be familiar in the treatment of the neonatus and the drowned, in the experience of others, and our own more fully established what was claimed for it at the first, reports were made in journals of larger circulation. Prof. T. Gaillard Thomas, M. D., of New York, refers to it approvingly in his paper in the *American Journal of the Medical Sciences*, of Philadelphia, on "A Century of American Medicine" (from 1776 to 1876). As it is now the property of the world, it is hoped it will be generally utilized at the various life-saving stations, as well as in *lying-in apartments*, in one of which it had its origin ten years ago.

